# Complete Genome Sequences of Extraintestinal Pathogenic Escherichia coli Clinical Isolates from Danish Ulcerative Colitis Patients

**DOI:** 10.1128/mra.01186-22

**Published:** 2023-01-30

**Authors:** Simon Meski, Carsten Struve, Hengameh C. Mirsepasi-Lauridsen, Andreas M. Petersen, Lotte Jelsbak, Ole Skovgaard, Karen A. Krogfelt

**Affiliations:** a Department of Science and Environment, Roskilde University, Roskilde, Denmark; b Department of Bacteria, Parasites and Fungi, Statens Serum Institut, Copenhagen, Denmark; c Department of Clinical Microbiology, Copenhagen University Hospital—Amager and Hvidovre, Copenhagen, Denmark; d Department of Gastroenterology, Copenhagen University Hospital—Amager and Hvidovre, Copenhagen, Denmark; e Department of Clinical Medicine, University of Copenhagen, Copenhagen, Denmark; f Department of Virus and Microbiological Special Diagnostics, Statens Serum Institut, Copenhagen, Denmark; University of Maryland School of Medicine

## Abstract

Extraintestinal pathogenic Escherichia coli (ExPEC) is a potential factor in ulcerative colitis etiology. We report here the complete genome and plasmid sequences of three Escherichia coli isolates, C 237-04 (p7), C 236-04A (p10A), and C 691-04A (p19A), obtained from fecal samples from ulcerative colitis patients in Copenhagen, Denmark.

## ANNOUNCEMENT

Ulcerative colitis (UC) is characterized by periods of colonic mucosal inflammation, including signs and symptoms such as diarrhea, rectal bleeding, and stomach pain, followed by periods of remission. The disease etiology remains unsolved, but several host and environmental factors have been implicated (1, 2), including an association with virulent and pathogenic Escherichia coli (3–6). Studying E. coli strains isolated from UC patients helps us understand their role in UC etiology. Three clinical E. coli strains were isolated from patients’ fecal samples as part of a Danish clinical case-control study on inflammatory bowel disease (IBD), with informed written consent from participants and permission from the Regional Ethics Committee for Copenhagen County Hospitals (permission number KA03019).

The E. coli strains were isolated by suspending feces in phosphate-buffered saline, plating them on SSI selective enteric medium (number 724; SSI Diagnostica, Hillerød, Denmark) (7, 8), and incubating the plates at 37°C overnight. The colonies were assessed visually as E. coli, the species were confirmed using a Minibact E kit (9), and the cultures were stored in glycerol at −80°C. DNA was purified from fresh cultures, incubated at 37°C overnight in LB medium, using the phenol-chloroform method as previously described (10). Separate DNA batches were prepared for short- and long-read sequencing. The purity and concentrations were measured using a NanoDrop One spectrophotometer.

Sequencing and postprocessing were performed as described by Lallement et al. (11) using a combined short- and long-read sequencing approach. The read length of long-read sequencing was favored by omitting shearing and size selection of DNA before library preparation using an SQK-LSK109 ligation kit and sequencing on an Oxford Nanopore MinION instrument with a R9.4.1 flow cell. Base calling of the fast5 files to fastq files was performed using Guppy v5.0.7, with the dna_r9.4.1_450bps_sup model. Reads with a length of more than 10,000 nucleotides (nt) were selected using Filtlong v0.2.1 (https://github.com/rrwick/Filtlong) and assembled into scaffolds using Flye v2.9 (12) with -g 5 –nano-hq arguments or with Minimap2 v2.24 Miniasm v0.3 (13) assembly and two iterations of Minimap2 Racon v1.5.0 polishing (MMR) (14). The Minimap2 settings used were -x ava-ont for assembly and -x asm5 for polishing.

Paired-end 150-bp short-read sequencing was performed by BGI Europe A/S on the BGISEQ-500 platform, and the reads were filtered and trimmed using fastp (15) with the quality filter settings -q 25 and -u 10. Hybrid assemblies from these scaffolds and short sequence reads were constructed using SPAdes v3.15, with the -isolate and -trusted-contigs settings (16), and Unicycler v0.5.0 (17). These hybrid assemblies further identified the small circular plasmids in C 237-04 and C 236-04A solely from short sequence reads; using Unicycler, all replicon sequences were rotated to begin with *dnaA* (for chromosomes) or *repA* (for plasmids). Sequence annotation was performed by NCBI using PGAP v6.3 (18). Default parameters were used for all software unless otherwise specified. Sequence and assembly statistics are presented in Table 1.

**TABLE 1:** Sequencing and assembly statistics

Isolate	Replicon	No. of reads		Coverage (×)		ONT *N*_50_ (bp)	Size (bp)	GC content (%)	No. of predicted CDS^*c*^	GenBank accession no.	SRA^*d*^ accession no.	
		BGI^*a*^	ONT^*b*^	BGI	ONT						BGI	ONT
C 237-04		11,632,828	41,675			25,238					SRR21857883	SRR21857880
	p7 (Chr)^*e*^	11,433,789		349	66		4,918,902	50.57	4,630	CP109921		
	pP7_1	49,975		1,203	0		6,230	49.31	8	CP109922		
	pP7_2	40,225		4,136	0		1,459	50.58	1	CP109923		
												
C 236-04A		11,648,518	8,522			29,467					SRR21857884	SRR21857881
	p10A (Chr)	11,521,884		347	13		4,987,582	50.85	4,734	CP109924		
	pP10A_1	27,678		1,010	0		4,109	45.44	5	CP109925		
	pP10A_2	22,567		1,097	0		3,086	46.01	4	CP109926		
	pP10A_3	16,005		978	0		2,455	48.80	4	CP109927		
	pP10A_4	12,099		1,464	0		1,240	46.13	1	CP109928		
												
C 691-04A		11,649,154	174,807			4,904					SRR21857885	SRR21857882
	p19A (Chr)	11,309,943		328	45		5,176,167	50.46	4,826	CP109929		
	pP19A_1	302,594		582	123		77,976	52.26	100	CP109930		

a BGI, BGISEQ sequencing; no. of reads mapped to each replicon using Bowtie2 v2.4.5 (19).

b ONT, Oxford Nanopore Technologies.

c CDS, coding DNA sequences.

d SRA, Sequence Read Archive.

e Chr, chromosome.

Serotyping and virulence characterization (7) were confirmed *in silico* using Web applications provided by the Center for Genomic Epidemiology, DTU (20), and EZClermont v0.6.3 (21). Isolates p7 and p19A were found to be genotypically more virulent than p10A, corresponding to the inflammatory stage of the patients from active and inactive colitis, respectively (7).

### Data availability.

This whole-genome sequencing project has been deposited at GenBank under accession number PRJNA882345. The version described in this paper is the first version. The accession numbers for the raw reads and assemblies are provided in Table 1.
